# ‘A Defence of Physick’: Thomas Reeve's proposed ‘fair experiment’ on the benefits of tar-water treatment (1744)

**DOI:** 10.1177/09677720251383856

**Published:** 2025-10-27

**Authors:** Max Cooper, Sarah Cooper

**Affiliations:** 1 Primary Care and Public Health, 12190 Brighton and Sussex Medical School, UK; 2 Department of Neurology, 156800 Royal Sussex County Hospital, Brighton, UK

**Keywords:** Controlled trial, James Lind, Bishop Berkeley, Hauksbee the younger, tar water, van Helmont, George Starkey, London, England

## Abstract

**Introduction:**

In 1744, the English physician Thomas Reeve (‘T.R.’, b.1700–1780) published two proposals for controlled trials of tar water treatment, a ‘universal’ medication promoted by Bishop George Berkeley (1685–1753).

**Methods:**

Qualitative analysis of historical medical texts.

**Results:**

In his ‘defence of physick’, Reeve questions Berkeley's clinical evidence and calls for a ‘fair experiment’ with a ‘competent number’ of patients (i.e. 20), half of whom should receive tar water. Participants should be ‘of the same Age, Sex, Constitution and Country, of the same way of living, and of the same Temperament’. Treatment must be ‘at the same Time and Place’, circumstances employed in Lind's 1747 scurvy trial. Reeve also proposes a simple two-patient trial to compare smallpox treatment with and without tar water.

**Discussion:**

It is tempting to see Reeve's proposals as an influence upon James Lind's 1747 scurvy trial. Evidence that could support this lies in Lind's consideration of both tar water and Berkeley's book within his 1753 treatise on scurvy. Likewise, Reeve's call to keep a ‘faithful register’ of clinical outcomes offers a possible link with Hauksbee the Younger's 1743 proposal for an 'experimentum crucis' of treatments for venereal disease. The contribution of Thomas Reeve to the development of controlled trials (including the ‘morality’ of withholding effective treatment) deserves greater recognition.

## Introduction

Thomas Reeve (1700–1780) was an English physician. He has no entry in the Oxford Dictionary of National Biography. Munk's roll of the Royal College of Physicians of London appears to be the only authority for Reeve's biography. Munk states:

Thomas Reeve, M.D., was born in Middlesex, and educated at Emmanuel college [see Figure 1], Cambridge, as a member of which he proceeded M.B. 1727, and M.D. 1732. He had studied physic for some time at Leyden under Boerhaave and Albinus, and was entered there 18th October, 1725. He was admitted a Candidate of the College of Physicians 22nd December, 1735; and a Fellow 29th December, 1736. He was Registrar from 1739 to 1741 inclusive; Censor in 1741 and 1749; Elect, 19th January, 1750; Consiliarius, 1751, 1752, 1753; and President from 1754 to 1763 included. Dr Reeve was elected physician to St Thomas's hospital [see Figure 2] in 1740, and resigned that appointment in 1760. Dr Reeve died at his house in Throgmorton-street [in London].^1^

**Figure 1.**
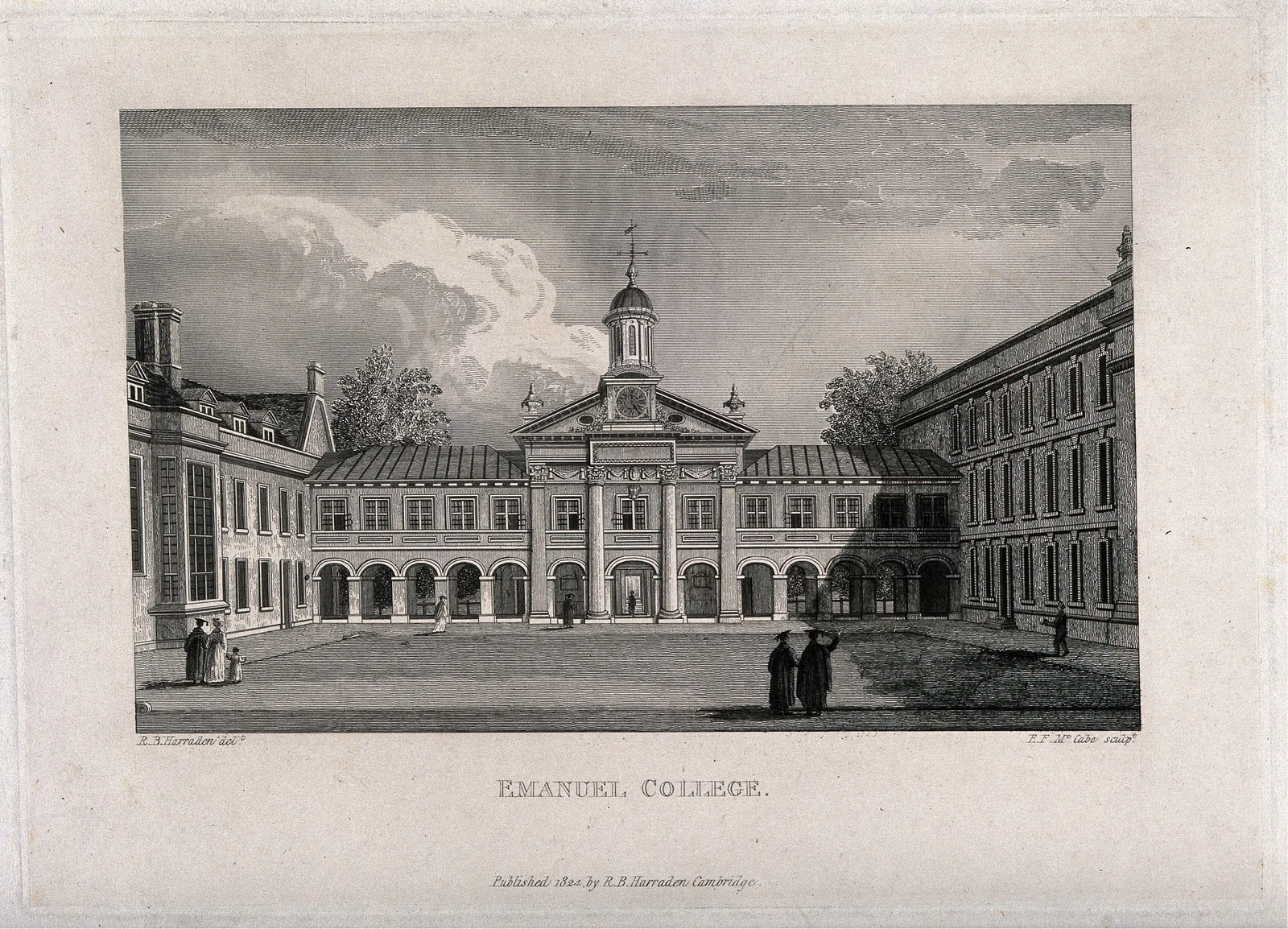
Emmanuel College, Cambridge: Front Court, showing the facade of the Chapel. Line engraving by E. F. McCabe, 1824, after R. B. Harraden. Image courtesy of the Wellcome Collection.

**Figure 2.**
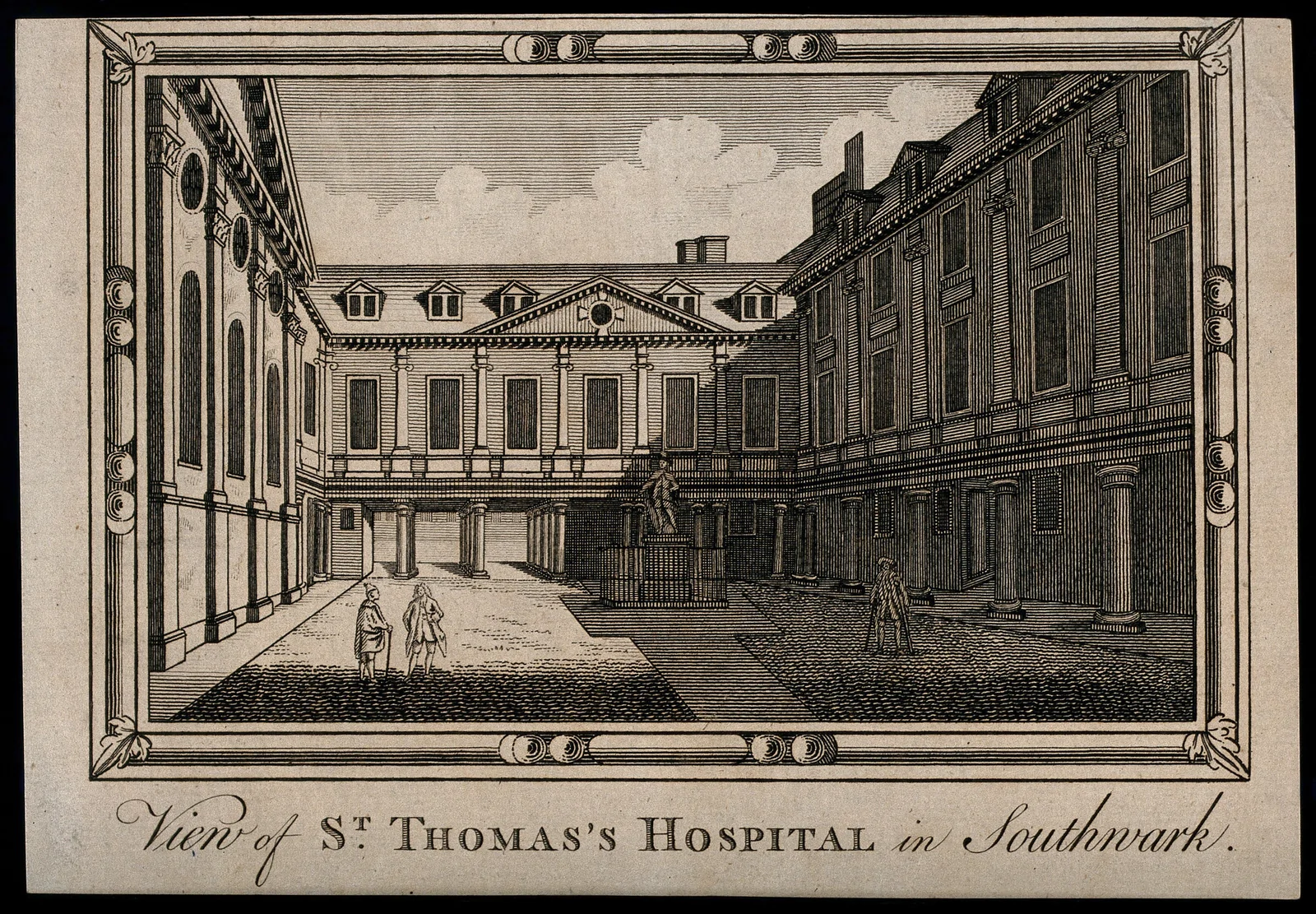
Old St. Thomas's Hospital, Southwark: Inside the first courtyard. Engraving. Image courtesy of the Wellcome Collection.

## Tar water

In his 1744 book *Siris*, George Berkeley (1685–1753, see Figure 3), Bishop of Cloyne, proposed tar water as a ‘universal’ medicine, that is, a panacea.^2,3^ *Siris* included clinical cases presented as evidence to support Berkeley's claims about the efficacy of the medication. Its publication triggered a paroxysm of interest in tar water across Europe and even into America.^3^ Berkeley's approach to treatment with tar water was summarised thus: ‘The stronger the better; provided the stomach can bear it’.^3^^(p2)^ Tar was produced from the resin of pine trees and Berkeley describes methods of its production, for example:

Put a gallon of cold water to a quart of [Norwegian tar, see Figure 4], stir and work them very strongly together with a flat stick for about four minutes. Let the vessel stand covered forty eight hours, that the tar may subside. Then pour off the clear water, and keep it close covered, or rather bottled, and well stopped, for Use.^2^^(p2)^

**Figure 3.**
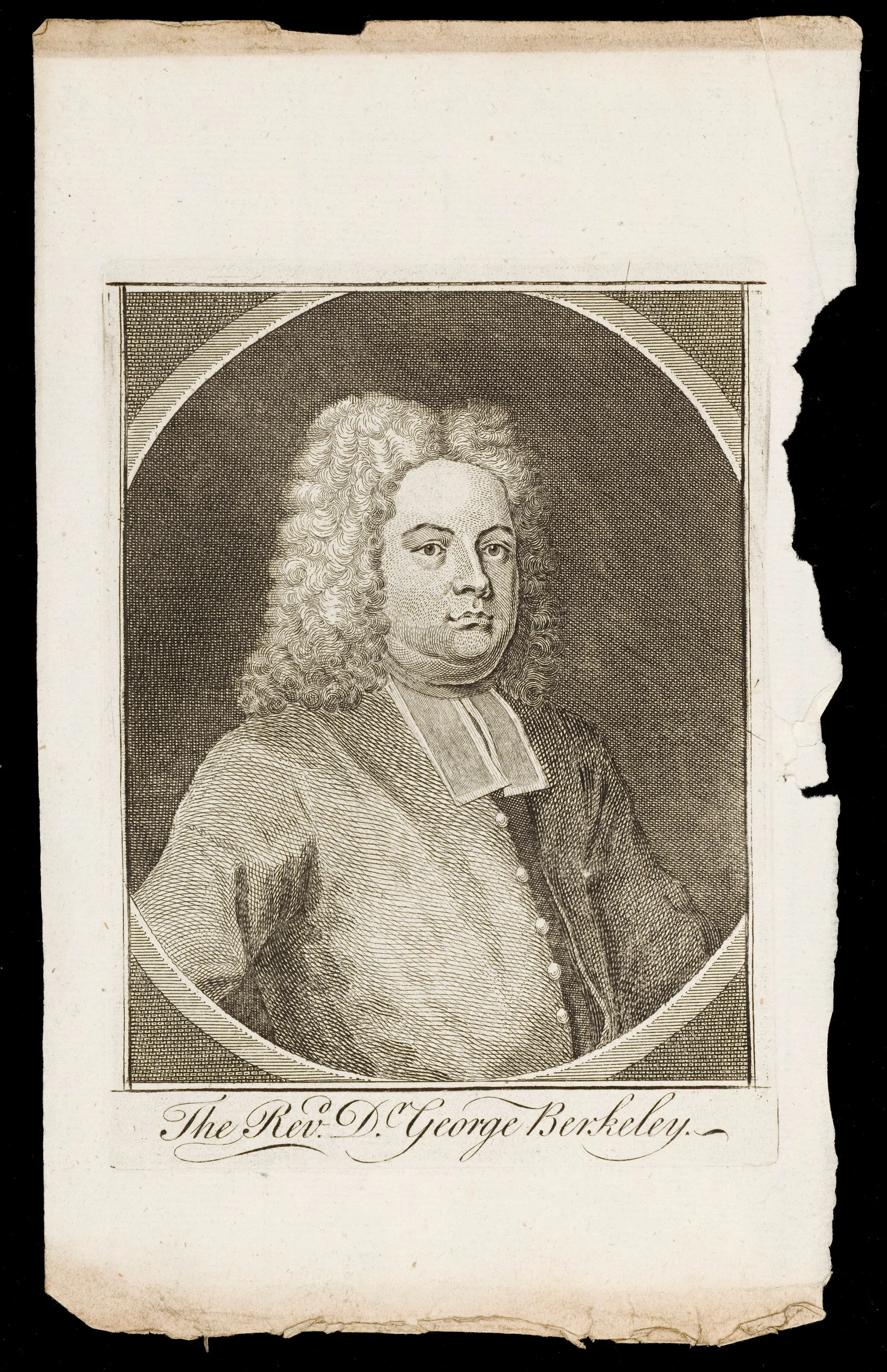
George Berkeley before he became a bishop, i.e. prior to 1734. Line engraving dated ca. 1759–1760. Image courtesy of the Wellcome Collection.

**Figure 4.**
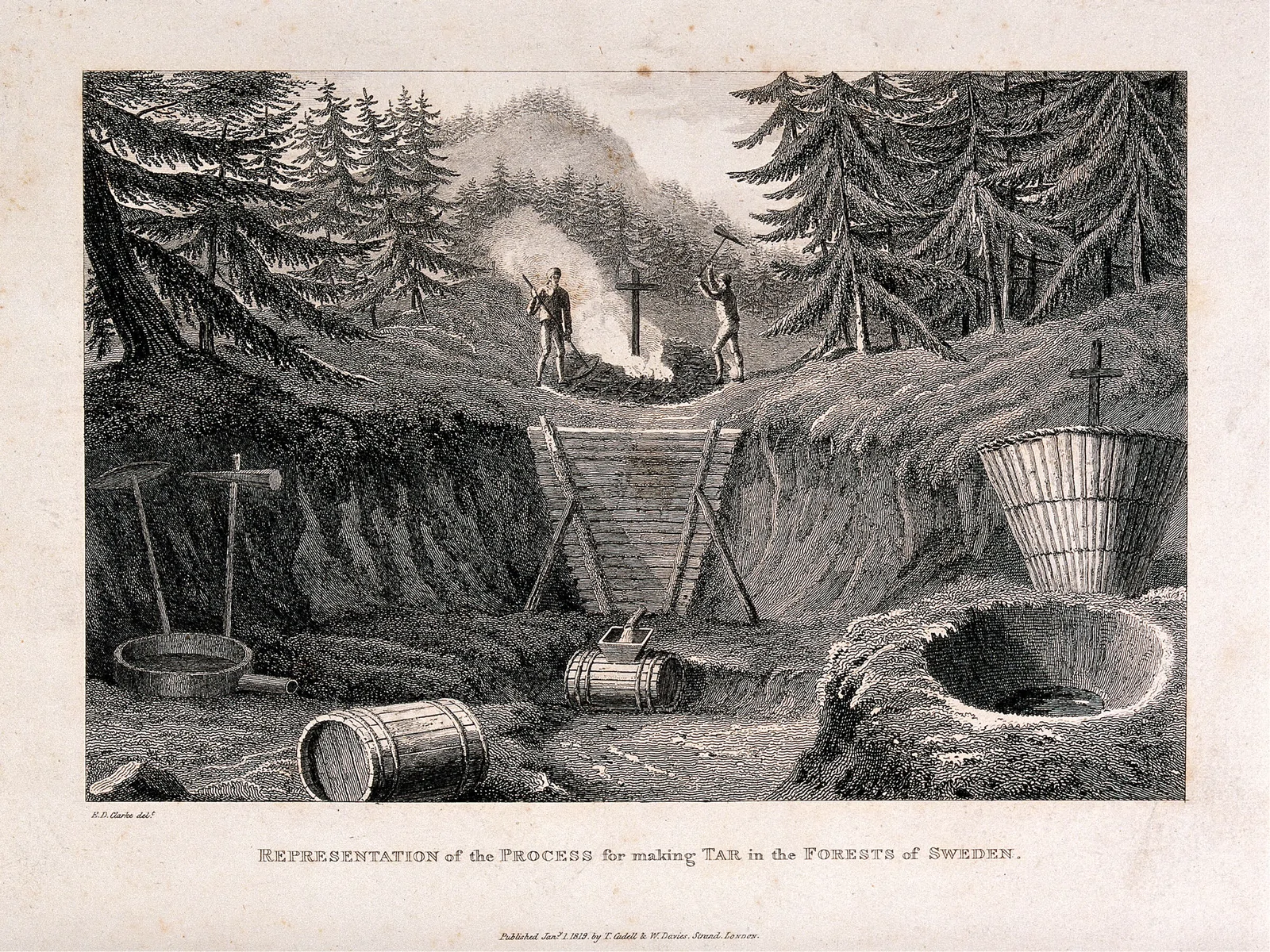
People making tar in the forests of Sweden. Etching after E. D. Clarke. Image courtesy of the Wellcome Collection.

## Thomas Reeve (‘T.R.’)

One overlooked aspect of the history of tar water lies in a 1744 book by ‘T.R.’ in response to Berkeley's claims in *Siris* and written ‘in defence of physic [i.e. the practice of medicine]’.^4^ This was published in London and entitled ‘A Cure for the Epidemical Madness of drinking Tar Water’.^4^ According to Munk, Thomas Reeve ‘was probably’ its author.^1^ There exists, however, written evidence from Reeve's own lifetime (and, notably, after Berkeley's death) confirming the former as the author ‘T.R’. This was published by Charles Alston in his 1770 ‘Lectures On The Materia Medica: Containing The Natural History of Drugs’.^5^ Alston initially sets out his stall by dismissing the existence of a ‘universal’ medicine:

It is scarcely worth while to enquire, whether there is such a thing as a panacea. … It appears not to be possible … Paracelsus and Helmont … [who] pretended [i.e. claimed] to the knowledge of such, used other means [i.e. treatments]. But what say you of his Right Reverend Lordship's [i.e. Bishop Berkeley] tar-water?…^5^

Alston then goes on to cite Reeve in his own rejection of tar water, specifically naming him as ‘T.R’:

[Tar water] is particularly recommended for the asthma: I know two men, who obstinately continued the use of it for a considerable time, without any benefit, till growing sensibly worse, they were obliged to give it over. Vid. [i.e. see] The cure of the epidemical madness of drinking tar-water. By T.R. (Thomas Reeve) M.D. London, 1744. 8vo. of 66 pages. ‘Your Lordship's genius, says he, is too sublime to stoop to useful and common virtues…’^5(p41)^

## Reeve's critique of Berkeley's clinical evidence

Reeve writes in a very deferential style in response to Bishop Berkeley. His rallying call in ‘defence of physic’ conveys a dissatisfaction with the state of ‘philosophical reasoning’ (i.e. scientific approach) in contemporary medicine.^4^ As part of this, he accuses Berkeley of ‘reducing the whole Materia Medica to a single Medicine’.^4^^(p6)^ Reeve also highlights a lack of evidence presented by Berkeley to support the deductions made from his ‘scanty Enquiry’ presented in *Siris*:

I must own [i.e. admit that] your Lordship sets out in such manner as if you really thought the Virtues of Tar-Water were to stand or fall by fair Experiment; but after having told us of a Variety of Cases, and prudently declined giving us a particular Detail of them, conscious, as it were, of the defect of such Evidence, ‘your Lordship drags in your philosophical Chain to eke out your scanty Enquiry’.^4^^(p12)^

Reeve then goes on to criticise the quality of Berkeley's evidence in specific ways:

You begin, my Lord, with telling us ‘that several were preserved from taking the Small Pox by the use of Tar-Water, others had it in the mildest manner, and others, that they might be able to take the Infection, were obliged to intermit [i.e. to refrain from] the drinking it [sic]’.^4^^(pp12–13)^

Reeve criticises Berkeley for not providing a specific number of cases. He also submits his own experience to show how clinical evidence can beget erroneous deductions:

two of my Patients were lately in such desperate Circumstances, that their Friends had almost determined to give them up to your Lordship's Water, when by an accidental Turn in their Distempers they both recovered. Now had they taken but one Dose of your Lordship's Medicine just before these critical Turns, it is probable your Lordship's learned Disciples, and past all doubt your unlearned ones would have given the Credit of their Cures to Tar-Water.^4^^(p14)^

Finally, Reeve refers to a recent epidemic where inducing vomiting constituted one treatment. He uses this to highlight a lack of clarity in Berkeley's clinical accounts, specifically the total number of patients (i.e. the denominator) who received the relevant intervention:

I must acquaint the Reader, that in this epidemical Fever of 1741 vomiting was found to be peculiarly serviceable; and whether this Operation [i.e. vomiting medication] might not cure twenty out of your Lordship's twenty five [cases of cure] we cannot possibly determine till your Lordship shall be pleased to tell us plainly the Number of those who had been vomited.^4^^(p34)^

## Reeve's proposal for a two group controlled trial

Reeve then sets out his own proposed experiment, starting with controlling for demographic differences between participants. By presenting this as ‘an universal Likeness’ between patients, he appears to be having a swipe at tar water. His proposed ‘experiment’ takes the form of a commentary on Berkeley's own method and is written in the third person and conditional tense, presumably from a sense of deep social deference:

Had you told us, my Lord, you had taken any Number of People of the same Age, Sex, Constitution and Country, of the same way of living, and of the same Temperament, in short in everything as much alike as was possible (and surely nothing less than such an universal Likeness is necessary to a fair Experiment)...^4^^(p15)^

He then points to the need for a comparison of a large number of participants who receive contemporaneous treatment under the same circumstances. It is of note that Reeve includes an untreated group, that is, one-half of the participants:

had your Lordship, I say, taken any competent Number of such Persons, and at the same Time and Place given Tar-Water to one half and not to the other.^4^^(p15)^

He does not state specifically whether the half who do not receive tar water should receive any other treatment. The implication is that they should receive no treatment at all. Finally, he calls for a ‘competent’ number of participants (citing twenty such cases) and the need to show a clear difference in outcome between the two groups:

and had your Lordship, after twenty such Experiments as carefully made, found that those who drank the Water were always either preserved from the Distemper or had it in a remarkably favourable manner, and that such as drank it not scarce ever escaped it, but had it like others in the same Circumstances, I should readily have allowed your Lordship to have some Grounds for your Conclusions.^4^^(p15)^

As part of his proposal, Reeve goes on to call for the keeping of a ‘faithful register’ of the experiment's results:

If your Lordship thinks fit to set up for an Experimenter in Physick [sic], surely you cannot think it below your Dignity to keep a faithful Register (as all Men who study any Branch of natural Knowledge ought to do) of all your Experiments…^4^^(p34)^

## Reeve's proposal for a two-patient controlled trial of tar water in smallpox

There is no evidence that Reeve ever undertook his proposed (or any other) controlled trial himself. This is surprising because, as a clinician, he clearly had the opportunity for direct comparisons of treatments. What is more, he specifically describes using tar water in two ‘desperate cases’,^4^^(p22)^ where he might have treated only one to allow for a basic comparison. In these two patients, he observed no improvement from tar water, so much so that it led to the ‘exasperating [of] both Cases to such a Degree as to oblige us to lay it aside’.^4^(^p22)^ Likewise, he proposes withholding treatment from two (i.e. rather than one of two) of his own patients with smallpox.^4^^(p16)^ Elsewhere, however, he describes a hypothetical scenario of this most basic of clinical trials, that is, two patients where only one is treated. It is of note that again he asserts the need for a fair comparison, for example, by insisting that the patients be from ‘the same house’:

Suppose, my Lord, two young sanguine Persons in the same House should be taken ill of the confluent Small Pox, and that one of these after having drank [sic] Tar-Water for three Weeks, according to your Lordship's Directions, to prevent the Infection, should recover, and the other, who had drank no Tar-Water should die: would your Lordship conclude from hence for the Efficacy of Tar-Water…^4^^(p16)^

## Parallels with Hauksbee the younger and James Lind

Reeve's proposal is of note for its place in the timeline of the development of the controlled trial of medical treatment, that is, as an unrecognised landmark between Hauksbee's proposal (1743) and Lind's landmark scurvy trial (1747). The close timing between Hauksbee, Reeve and Lind and their lack of participant selection by randomisation hints at a common source. This is in contrast to the seventeenth-century proposals of van Helmont^6^ and George Starkey,^7^ both of whose methodologies included the drawing of lots. Like those two, Reeve's proposal sets out a simple split into two groups. Hauksbee's and Lind's models, by contrast, provided for more than two groups^8^ and Reeve's notion of including an untreated group is not recapitulated in Lind's trial. Presumably this was because Lind was familiar with the natural history of untreated scurvy in sailors.

Reeve's proposal is set out in some circumlocution, a style that is in stark contrast with the ten practical steps of Hawksbee's proposed ‘experimentum crucis’ of treatments for venereal disease.^8^ One aspect of Reeve's proposal suggests that he was influenced by Hauksbee, namely his call for a ‘faithful register’ of the results of experiments. That Hauksbee was considered in this wider debate about tar water is evident in another contemporary book on the subject written by ‘Philanthropus’:

But pray, Sir, if the Theory of Medicine ‘be deem'd vain babbling’, … wherein consists the Difference of an upstart Quack, educated under Rock, and a rational Practitioner? the one will tell you of the many Wonders wrought by his Nostrums, and possibly (follow Hawksbee [sic]) challenge the World with his Experimentum Crucis…^9(p5)^

It is likely that Reeve's book was a further stimulus (i.e. in addition to Hauksbee) for Lind for two reasons. First, because Lind reveals in his 1747 treatise that he examined Berkeley's book *Siris*.^10^ What is more, Lind clearly dabbled with tar water himself: ‘I have myself experienced … tar-water…’^10^^(^^p185)^ and ‘In extremity tar-water may be tried, fermented … by which it will certainly become much more antiscorbutic’.^10^^(p224)^

It is, therefore, very likely that Lind read much more widely about tar water, probably including both Reeve's book and Berkeley's response to it.^11^ The latter states:

Since the former Edition of this Pamphlet the Bishop has been attacked by another Physician, in a Piece entitled, A Cure for the Epidemical Madness of drinking Tar Water, by T.R. M.D. from whose Judgment of this Medicine we learn that Doctors differ as well as Divines.^11^^(p31)^

Second was Reeve's call for participants to be studied ‘at the same Time and Place’. This was, famously, the case for Lind's participants:

Their cases were as similar as I could have them. … They lay together in one place, being a proper apartment [sic] for the sick in the fore-hold [of HMS Salisbury at sea]; and had one diet common to all.^10^

## Reeve and the ‘morality’ of withholding effective treatment

In contrast to his call to withhold treatment from half of the patients as part of a fair trial, Reeve makes an astute comment on the ‘morality’ of omitting interventions known to be effective. Here, Reeve appears to be referring to his faith in ‘blisters’ (cupping) as an effective treatment of pleurisy:

Suppose then, my Lord, we already know a Method which has been found upon an infinite Number of Trials to cure ninety nine persons out of a hundred, of such as apply [i.e. come for treatment] early to us in this dangerous Distemper, does your Lordship think any honest Man can conscientiously depart from such a Method for the Sake of trying what in his own Judgment must be hurtful, at least inefficacious. … Such a Method I can assure your Lordship we now have… (p. 25)

## Thomas Reeve, the physician

Through his presidency of the Royal College of Physicians of London, Reeve was clearly an establishment figure held in high esteem by his medical peers. Indeed, his book may constitute as much a defence of London's College of Physicians (and its members’ revenue) as a defence of physic itself. In his book, he comes across as an experienced, deferential and open-minded physician. The lattermost trait is evinced in his opinion that ‘I think Tar-Water no contemptible Medicine in some Cases’.^4^^(pp21–22)^ This is in contrast to many doctors of his day who rejected outright new treatments promoted by medical peers.^8^ Despite acting ‘in defence of physic’, it is clear that some of Reeve's own medical treatments were antiquated and his practice included interventions potentially more dangerous than tar water. This is most notable in his favourable opinion on bloodletting. This observation is ironic given that similar trials to *disprove* the value of bloodletting had been proposed by van Helmont (1648) and Starkey (1658). Reeve reveals his deep faith in bloodletting with regard to the treatment of ‘pleurisie’:

I have had, my Lord, such ample Experience of this Matter, that I must believe nothing so effectual as repeated bleeding to prevent immediate Death or a Consumption…^4^^(p26)^

Ironically, this is in contrast to Berkeley. The latter notes the following opinion, predictably a reflection of his deep faith in tar water:

In peripneumonies and pleurisies I have observed tar-water to be excellent, having known some pleuritic persons cured without bleeding, by a blister early applied to the stitch, and the copious drinking of tar-water.^2^^(p37)^

## Conclusion

The life of Bishop Berkeley and the impact of his tar water on patients, doctors and society has been examined in detail elsewhere.^3^ He was probably persuaded of the benefits of tar water due to its herbal origins, bitter taste and personal experience of observing its use. Tar water may have brought some clinical benefits through hydration, the placebo effect and avoidance of other treatments used at the time, such as mercury. Berkeley's grand claims in favour of tar water treatment stimulated wide debate over clinical evidence, a controversy exemplified by his insightful retort: ‘Who shall decide when the doctors disagree!’.^9^ Despite the wealth of clinical evidence that exists today, this remains an enduring question. It is particularly relevant to the formation of modern medical students who must learn to practise ‘off piste’ in situations where formal clinical guidelines cannot be applied. This is commonly the case in general practice (i.e. family medicine).

Reeve's proposed experiment seems to have had little impact on the evaluation of tar water. This is likely to be because Reeve does not seem to have undertaken the very controlled comparisons that he called for. As such it could constitute little more than a rhetorical device to undermine Berkeley's grand claims for tar water. Ultimately, Reeve may have won Berkeley over to his controlled method of comparing treatments (if not to his low impression of the value of tar water). This is hinted at in Berkeley's own proposal for a controlled trial of tar water treatment from 1752, which bears striking methodological similarities. Here, Berkeley's proposal calls for tar water to be tested in two patient groups living under identical circumstances. Nevertheless, we suggest that Berkeley's brevity, imitation of Reeve's methodology and choice of flippant language lays bare the substance of this proposal as a rhetorical device for persuading his audience:

The Experiment may be easily made, if an equal Number of poor Patients in the small-pox, were put into two Hospitals at the same Time of the Year, and provided with the same Necessaries of Diet and Lodging, and, for further Care, let the one have a Tub of Tar-Water and an old Woman; the other Hospital, what Attendance and Drugs you please.^12^

The present article offers new evidence of the contribution of Thomas Reeve to the development of the controlled trial of medical treatments. It is surprising that Reeve describes undertaking no such comparisons himself, given that he clearly used tar water in some of his own patients and had plenty of opportunity to do so. Reeve's antiquated beliefs about treatment (e.g. cupping and bloodletting) serve as a reminder to modern doctors that even those clinicians who are passionate about evidence-based medicine remain subject to bias, particularly in regard to the outcomes of their own treatment. This distortion in clinical judgement surely arises from the significant financial rewards that have historically accrued to doctors from their own treatments. In contrast to his beliefs about outdated medical treatments, Reeve's proposed method for studying tar water reveals significant critical thought.

Reeve's book is particularly notable for its call for ‘sufficient evidence’,^4^^(p61)^ and emphasis on a ‘fair’ comparison, that is, endeavouring to compare like with like. The chronology of publications by Hauksbee (1743) and Reeve (1744) suggests a confluence of critical ideas about comparing treatments in the years shortly before Lind's groundbreaking scurvy study of 1747. The life of Thomas Reeve, MD, and his contribution to the development of controlled trials (including the ethics of withholding treatment considered to be effective) deserve greater recognition.
